# The factors affecting the performance of the tunnel wall drilling task and their priority

**DOI:** 10.1038/s41598-024-60381-3

**Published:** 2024-04-26

**Authors:** Peng-Fei Gao, Jin-Yi Zhi, Ji-Dong Hu, Jin Wang, Yong-Sheng Xu, Rui Zou, Tie-Cheng Ding, Lin Yang

**Affiliations:** 1 Department of Industrial Design, School of Design, Southwest Jiaotong University, Chengdu, 610031 China; 2 China Railway Eight Bureau Group Electrical Engineering Co., LTD, Chengdu, 610500 China; 3 Department of Industrial Design, School of Mechatronic Engineering, Southwest Petroleum University, Chengdu, 610500 China; 4 School of Design, Southwest Jiaotong University, Chengdu, 610500 China

**Keywords:** Tunnel wall drilling, Task performance, Man–machine environment, Priority factors, Mechanical engineering, Civil engineering

## Abstract

Clarifying the relationship between the man–machine environment and its impact on the tunnel wall drilling task performance (TWDTP) is crucial for enhancing the task performance. Based on a questionnaire survey, indicators of the man–machine environment that affect the TWDTP were proposed in this study, and exploratory factor analysis and a structural equation model were employed to examine the potential factors influencing the task performance and their degrees of influence. By comparing the discrepancy between the perceived performance and importance, the satisfaction of potential factors was evaluated, and the priority order for optimizing these factors was determined by considering the degree of influence and dissatisfaction. The results of survey data analysis based on actual tunnel drilling operation scenarios indicated that tools had the greatest impact on the TWDTP, followed by the quality of the physical environment, while human factors had the least influence on the task performance. Convenient functional maintenance is the key to improving the TWDTP, along with enhancing the quality of the working environment. Once these main aspects are optimized, it is important to consider additional factors such as availability of spare tools, efficient personnel organization, man–tool matching, and safety and health assurance. This research approach provides significant guidance in understanding the relationships between the man–machine environmental factors affecting the performance of complex engineering tasks and identifying key influencing factors, thus providing essential insights for optimizing the TWDTP.

## Introduction

The operating mileage of the metro in China has exceeded 9000 km, with only an additional 1442 km planned for 2022. The installation of cables, communication, and signal optical cables inside the tunnel is a crucial aspect of subway tunnel construction, requiring numerous holes to accommodate cable supports. On average, each 21 km of metro tunnel necessitates a total of 620,000 installation holes of various sizes. However, the current tunnel wall drilling task still relies on handheld tools for operation. As the tunnel wall drilling task is a short-term phased task in tunnel construction, it is difficult to train and maintain a large-scale skilled drilling workforce. Meanwhile, in order to mitigate the impact of metro construction on urban road congestion, tunnel drilling tasks are often required to achieve higher efficiency for shortened construction times. Therefore, it is important to improve the tunnel wall drilling task performance (TWDTP). However, compared to above-ground tasks, the underground space drilling task is more challenging and uncomfortable for personnel^1^. The underground construction environment creates potential safety risks, noise, dust, vibrations, and other environmental issues. The use of handheld high-power electric pneumatic hammer drills further complicates tunnel wall drilling^2^. In addition, research has indicated that the efficiency of task organization^3^, ergonomic design of tools, and use of functional aids^4–7^, as well as the physical environment and safety climate, also affect the TWDTP. Considering the diversity of factors that affect the TWDTP and the potential correlations between factors, it is difficult to improve the TWDTP. Therefore, it is necessary to determine the factors affecting the TWDTP and identify factor priorities to provide key objects for improving the TWDTP.

Individuals are responsible for carrying out drilling tasks. Their working posture, operational behavior, and repetition frequency can result in fatigue and discomfort^3,8,9^, as well as potential musculoskeletal disorders and soft tissue damage^10^. The working posture while holding high-power tools weakens the joint strength of the human body and increases the uncertainty and task completion time^11^. Research has shown that completing drilling tasks while fatigued reduces the required time but increases the error rate^12^. Furthermore, fatigue diminishes muscle capacity and impairs movement control and accuracy^13^. Training and management can improve organizational performance, and teamwork is also closely related to task performance^14^. Task planning, such as equipment commissioning, equipment handling, hole cleaning, equipment and personnel moving, and setting the next hole, will also impact the operation time and task performance^15^.

Tools are essential in manual work, and the functional integrity of tools is the most basic requirement for completing tasks. Drilling tasks on reinforced-concrete structures accelerate bit wear, cause electrical component failure in tools, and decrease the drilling efficiency^16^. Therefore, convenient part replacement methods and scientific bit replacement schedules are necessary^17^. When conducting drilling operations on reinforced-concrete buildings, using electric pneumatic hammer drills and other tools requires significant physical strength^18^. Therefore, the performance of tools and man–machine adaptability directly affect the execution efficiency of tasks. For example, Sasikumar et al.^19^ discovered that handheld drilling machine vibrations influence the skeletal muscle force within the arm and alter the contact force between the handle and the hand, which is influenced by factors such as the handle shape and hand posture. Rempel et al.^20^ found that the control performance of hammer drilling tools, such as how users realize ergonomic control of the feed force, feed direction, lateral force, and other factors, also affect the drilling efficiency. The development and use of auxiliary tools is also of great importance for reducing the physical load on the body applying the force and improving the drilling efficiency. Alabdulkarim et al.^1^ and Kim et al.^5^ proposed the use of arm support exoskeletons as auxiliary means to assist with drilling tasks. Exoskeletons have shown significant effectiveness in reducing muscle activity, discomfort, and drilling error rates. However, these exoskeletons may increase the load and discomfort in other body parts while limiting movement flexibility^21,22^.

The working environment comprises the physical surroundings and safety climate. In underground space construction, the physical environmental characteristics, such as darkness, noise, dust, air quality, and construction waste, significantly impact operations. Operators handling handheld electric hammer drills are exposed to forces, torques, vibrations, noise, and silica dust^17,20^. When the drill encounters a reinforcement, the vibration level of the drill handle increases, affecting the stability of worker operations and leading to hole position deviations, thereby reducing the effective hole positioning rate^18,23^ and potentially resulting in drill bit fracture, compromising operational safety^15^. The safety climate refers to operators' understanding of workplace safety measures (including policies, procedures, and practices) and their adjustment of their behaviors accordingly^24,25^. High work pressure and potential safety risks diminish operators' perception of the environmental safety, increase stress, and can lead to fear, depression, and job burnout^26,27^. Research has found that individuals with a higher perception of the external environmental security exhibit more positive safety attitudes and behaviors^24,28^. However, long-term fear, fatigue, and pressure to meet deadlines will make individual behavior unstable and unpredictable^29^, thereby affecting operators' attitudes toward production and task performance^25^. The influence of the safety climate on the safety behavior and outcomes in underground space engineering construction has been widely studied and considered^28,30^.

Productivity is an important standard for evaluating man–machine environmental systems^6^. It quantifies the relationship between task completion and the time taken to accomplish it. The measurement indicators of drilling productivity include the rate of penetration, i.e. drilling meter/second^31^, and the hole position quality, i.e. the deviations between the target position of the hole and the bit position, as well as the deviations in the depth direction^4^. In addition, the loss of a cemented carbide bit will reduce the drilling efficiency, while replacing the alloy bit will increase the drilling cost and additional time consumption^15^. In subway construction, there is a high requirement for productivity, as the project tasks need to be completed within a shorter timeframe. This is necessary to minimize the disruption caused by the construction on the normal urban order. Consequently, a higher drilling efficiency, higher hole quality, and reduced consumable loss serve as crucial indicators for measuring the performance of tunnel wall drilling tasks.

The underground engineering construction environment is complex, with various interactive human–machine environmental factors affecting the task performance. However, most studies currently treat humans, tools, and the environment as separate entities^32,33^, lacking an understanding of their correlations. The relationships between these factors and their impacts on the task performance are intricate. For instance, poor ergonomic tool design can lead to personnel injuries, resulting in increased personnel replacement and training costs. Additionally, task interruptions and reduced production times decrease the efficiency, further influencing the task performance, which can be exacerbated by adverse environmental conditions^34^. The complexity of these relationships adds to the research challenge. Nevertheless, by investigating the impacts of these factors on the performance and assessing their performance contributions within tasks, we can determine the priority order for optimizing factors to enhance the performance. This study focused on a realistic scenario of tunnel wall drilling tasks to explore the degrees of influence and satisfaction of human–machine environmental factors on the task performance through a questionnaire survey and data analysis. The specific research objectives include the following:

1. Identifying the key man–machine environmental indicators that affect the tunnel wall drilling task performance.
2. Utilizing exploratory factor analysis to extract potential factors affecting the drilling performance and employing structural equation modeling to quantify the relationships between these factors and their impact on the TWDTP.
3. Conducting a comparative evaluation of the perceived performance and importance to assess satisfaction with potential factors.
4. Determining the factor priority to optimize potential factors based on a comprehensive consideration of their degrees of influence and dissatisfaction.

## Method

### Questionnaire design

#### Evaluation questionnaire

To assess the impact of the man–machine environmental indicators on the TWDTP, an evaluation questionnaire was designed based on the literature findings, including factor selection^9,16,17^, measurement item construction^6,35^, and psychometric tools^36^. The evaluation questionnaire comprised two parts. The first part was a scale to measure drilling worker’s perception of the indicators that affect the TWDTP. Indicators were identified from three perspectives: humans, tools, and the working environment. We consulted construction project managers, researchers specializing in human–machine efficacy, and subway tunnel drillers to ensure the indicators were comprehensive and reasonable. The refined indicators were used to construct the items of the perceived performance evaluation scale for drilling tasks. The Likert scale was used to quantify the perceived performances of the indicators using a five-point scoring method, ranging from 1 to 5 points, with 1 denoting "very inconsistent" and 5 denoting "very consistent." The second part of the questionnaire measured the tunnel wall drilling task performance. The three questions were used to record the task performance of the drilling workers who completed the first part of the questionnaire in the actual drilling task. encompassing the total number of boreholes completed within a two-hour timeframe^31^, the count of effective holes^4^ (meeting the requirements for the aperture radius, depth, and offset simultaneously), and bit loss quantity^15^.

#### Indicator importance evaluation scale

To evaluate the importance of human–machine environmental indicators that affect the TWDTP, an indicator importance evaluation scale was designed using the same items as the indicator perception performance scale^37^. The indicator importance scale adopted the Likert five-point scale, in which 1 meant "very unimportant" and 5 meant "very important." At the end of the scale, no questions were set to record the performances of drilling tasks.

#### Indicator reliability and validity analysis

The indicator perceived performance evaluation scale was used to conduct a trial survey. Based on the results of the trial survey, the reliability and validity of the scale were analyzed^38^. The reliability of the question for a certain indicator was examined by comparing the rise and fall in the Cronbach’s α value after deleting certain indicators^39^. The Cronbach’s α is usually used to reflect the internal consistency of multiple indicators; the higher the consistency is, the higher the indicator reliability is. The Cronbach's α coefficients for humans, tools, and the working environment exceeded 0.7, suggesting strong internal consistency within each domain. Furthermore, the Cronbach's alpha coefficient was also above 0.7 for the total scale, indicating a high overall consistency. Factor analysis was used to analyze the structural validity of the scale^40^. The Kaiser–Meyer–Olkin (KMO) values of humans, tools, and the working environment exceeded 0.7, and the *P*-value of Bartlett’s test was less than 0.05, indicating that the scale had good structural validity. In addition, expert validity analysis^41^ was used to invite construction project managers and human–machine efficacy researchers to judge the validity of the three measured items of humans, tools, and the working environment. The results showed that measured items had expert validity. SPSS Statistics 22 was used to analyze the reliability and validity of the trial survey data. Consequently, we identified 27 indicators that potentially influence the tunnel wall drilling task performance, as summarized in Table 1.

**Table 1:** Reliability and validity analysis of indicators affecting drilling task performance.

Variable	Indicator	Cronbach’s alpha	KMO	Bartlett’s test (P)	Number of indicators
Human	Teamwork	0.807	0.709	0.000	7
	Clear assignment of tasks				
	Operation training				
	Personal safety awareness				
	Personal responsibility consciousness				
	Drilling experience				
	Engineering construction experience				
Tool	Fault and overload warning	0.847	0.740	0.000	9
	Use safety				
	Sufficient protective tools				
	Easy replacement of parts				
	Sufficient vulnerable parts				
	Man-aided design				
	Man–machine adaptive design				
	Sufficient equipment types				
	Intact function and appearance				
Environment	Less dust	0.845	0.712	0.000	11
	Clean construction site				
	Good air quality				
	No noise hazard				
	Bright light				
	Emergency plan and drill				
	Sufficient protective materials				
	Injury report and timely treatment				
	Reasonable safety system				
	Reasonable project schedule				
	Safety inspection and hidden danger investigation				
Total scale		0.920	–	–	27

### Participants and surveys

Since women are often absent from the manual work in underground construction, we invited male drillers with more than 1 year of experience to participate in the survey through the engineering management agency. After informing them about the survey tasks and procedures, participants were selected by voluntary participation. Eligible participants were required to have been engaged in tunnel wall drilling tasks in the past month and to not have suffered muscle or bone injuries. Finally, 40 male workers aged 25 to 48 (average age 36) participated in the survey. We confirm that all the participants provided appropriate informed consent and details through face-to-face conversations. Additionally, we confirm that this research received approval from the ethics committee of Southwest Jiaotong University. At the same time, we confirm that all research was performed in accordance with relevant regulations.

**Figure 1:**
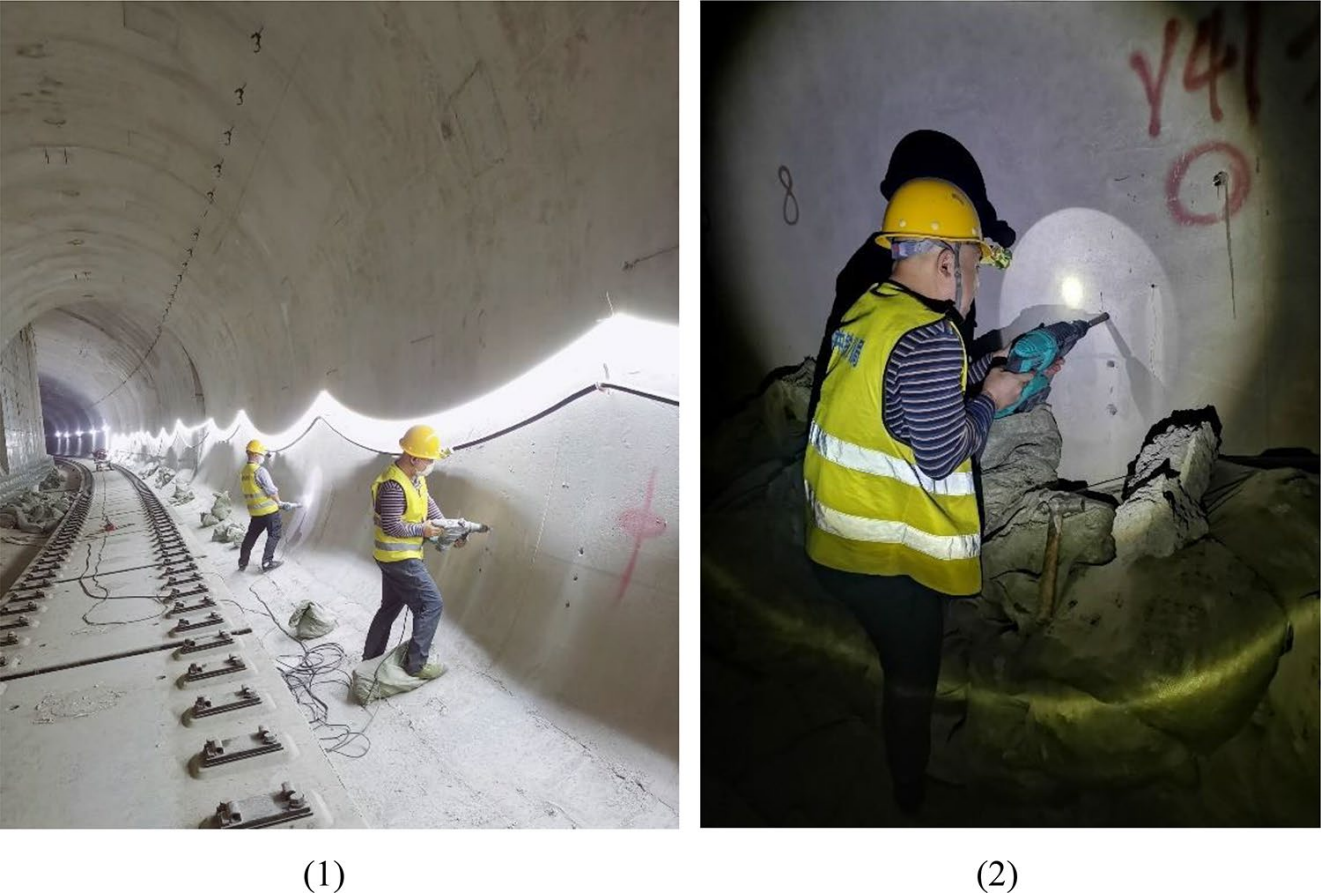
Drilling operation scenario: (**1**) Drilling environment (pictures were overexposed) (**2**) Drilling behavior.

To ensure the authenticity and reliability of the survey results, we conducted an investigation within a real engineering task. The survey consisted of three main steps. First, prior to the drilling operation, participants completed an indicator importance scale with the assistance of the staff. Subsequently, the participants carried out a 2-h drilling task. They drilled holes with diameters of 12 mm and depths of 80 mm at three designated mark points, spaced radially at intervals of 130 mm and situated at heights ranging from 600 to 860 mm above the ground. The survey scenario is shown in Fig. 1. It should be noted that the drilling task in the survey was part of the project schedule and not an additional task solely for the purpose of the survey. Finally, at the end of the two-hour period, participants completed an indicator perception performance scale with the assistance of the staff, while the project management personnel recorded the number of boreholes, the number of effective holes, and the number of bit losses that occurred within the given timeframe.

### Data processing

#### Analysis of potential factors affecting drilling task performance

##### Extraction of potential factors

In order to facilitate the respondents' clear understanding of the items, the variables related to humans, tools, and the environment that may affect the TWDTP were refined into 27 indicators, but this also increased the complexity of the relationships between the variables. Exploratory factor analysis was used to reduce the dimension of the 27 indicators and extract the potential factors affecting the TWDTP. SPSS Statistics 22 was used to perform exploratory factor analysis. Principal component analysis was used to extract potential factors, and the initial eigenvalues having values ≥ 80% was used as the extraction standard of the potential factors.

##### Conversion of potential factors to measured variables

According to the corresponding relationships between potential factors and indicators, the indicator importance evaluation result was taken as the weight (formula (1) below), and the potential factors were converted into measurement variables through the weighted summation of the indicator's perceived performance evaluation (formula (2) below), providing data for the study of the impact of potential factors on the performance.

*N* represents potential factors, and A represents an indicator. It was assumed that the potential factor n contained y indicators, i.e. n = (A1, A2, A3, …, Ay). The weight Q of the indicator ay was the dimensionless expression of the importance evaluation Z of the indicators contained in the potential factor n:

1 $${Q}_{{A}_{y}}=\frac{{Z}_{{A}_{y}}}{{\sum }_{y}{Z}_{{A}_{y}}}.$$

According to the inclusion relationship between indicators and potential factors and their corresponding weights, the perceived performance evaluation value of the potential factors in each questionnaire (the perceived performance evaluation value of potential factor N in the t-th questionnaire) was calculated to form the perceived performance evaluation data of potential factors ${P}_{tN}$:

2 $${P}_{tN}={\sum }_{y}\left({P}_{t{A}_{y}}\cdot {Q}_{{A}_{y}}\right).$$

#### Analysis of the impact of potential factors on performance

A structural equation model was employed to analyze the relationships between individuals, tools, and the working environment, as well as determine their impact on the TWDTP. Three measurement models were constructed: one for individuals and their potential factors, one for tools and their potential factors, and one for the working environment and its potential factors. Subsequently, a structural model was established to describe the relationships between individuals, tools, and the working environment, including the path structure of their impact on drilling task performance. The measurement and structural models were integrated, and the hypothetical model diagram was generated using the Amos graphics software. The maximum likelihood method was applied to fit the hypothetical model diagram with the evaluation data of the perceived performance and the performance data of potential factors. The Amos graphics facilitated the model fitting process.

#### Indicator satisfaction evaluation

Satisfaction was determined by comparing the perceived performance (*G*) of a product or service with its importance (*Z*). If the perceived performance met or exceeded the importance, it was defined as satisfaction; otherwise, it was considered dissatisfaction. Additionally, the absolute value of the ratio of the difference between the perceived performance and the importance (*G* $-$ *Z*) to the importance indicates the degree of dissatisfaction (*N*) in the product or service experience^37^. The comparative evaluation method of the perceived performance and importance was employed to assess whether the indicators affecting the TWDTP fulfilled the requirements of drilling workers regarding personnel, tools, and environmental conditions. If the perceived performance of an indicator surpassed its importance, it indicated satisfaction. Conversely, if the perceived performance was lower than the importance, it signified dissatisfaction and highlighted the need for improvement. The degree of dissatisfaction (*N*) reflected the priority of enhancing the indicator^37^.

3 $$N=\left|(G-Z)/Z\right|.$$

#### Priority determination of potential factor optimization

The influences of potential factors on the TWDTP and the dissatisfaction of factors were comprehensively considered to determine the key points to improve the task performance. Factors with a greater impact on the performance and higher dissatisfaction levels should be prioritized for improvement. Conversely, factors with lower impacts on the performance and lower dissatisfaction levels should have lower priority for improvement.

## Results

### Potential factors affecting drilling task performance

The exploratory factor analysis results (Table 2) indicated that personnel organization efficiency, safety responsibility awareness, and construction experience are potential human-related factors influencing the TWDTP. These three factors collectively accounted for 85.467% of the variance in human-related indicators. In addition, the reliability test of potential factors was carried out in SPSS Statistics 22. The values of the internal consistency index, i.e. Cronbach’s α, of these three potential factors were 0.859, 0.842, and 0.721, respectively, indicating good reliability of these extracted factors.

**Table 2:** Analysis of potential human related factors affecting drilling performance.

Indicator	Potential factors		
	Personnel organization efficiency	Safety responsibility awareness	Construction experience
Teamwork	0.864		
Clear assignment of tasks	0.859		
Operation training	0.837		
Personal safety awareness		0.909	
Personal responsibility consciousness		0.890	
Drilling experience			0.929
Engineering construction experience			0.754

Factor extraction standard: initial eigenvalues ≥ 80%, extraction method: principal component analysis.

Table 3 presents potential factors associated with tools that impacted the drilling task performance, namely the safety performance, convenient maintenance, man–tool matching, and availability of spare tools. These four factors collectively accounted for 82.807% of the variance of the tool-related indicators. The values of the internal consistency index, i.e. Cronbach’s α, for these four potential factors were 0.909, 0.817, 0.634, and 0.436, respectively, indicating good reliability of these extracted factors.

**Table 3:** Analysis of potential factors affecting drilling performance related to tools.

Indicator	Factor			
	Safety performance	Convenient maintenance	Man–tool matching	Availability of spare tools
Fault and overload warning	0.930			
Use safety	0.862			
Sufficient protective tools	0.844			
Easy replacement of parts		0.837		
Sufficient vulnerable parts		0.731		
Manual man–tool matching			0.817	
Man–machine adaptive design			0.762	
Equipment types sufficient				0.675
Intact function and appearance				0.955

Factor extraction standard: initial eigenvalues ≥ 80%; extraction method: principal component analysis.

Table 4 provides an overview of potential environment-related factors that impacted the drilling task performance, namely the physical environment, safety and health guarantee, and management. These three factors collectively explained 80.593% of the variation in environmental-related indicators. The values of the internal consistency index, i.e. Cronbach’s α, for these three potential factors were 0.885, 0.778, and 0.833, respectively, indicating good reliability of these extracted factors.

**Table 4:** Analysis of potential factors affecting drilling performance related to environment.

Indicator	Component		
	Physical environment	Safety and health guarantee	Management
Less dust	0.908		
Clean construction site	0.827		
Good air quality	0.812		
No noise hazard	0.791		
Bright light	0.728		
Emergency plan and drill		0.927	
Sufficient protective materials		0.882	
Injury report and timely treatment		0.695	
Reasonable safety system			0.898
Reasonable project schedule			0.824
Safety inspection and hidden danger investigation			0.634

Factor extraction standard: initial eigenvalues ≥ 80%, extraction method: principal component analysis.

### Relationship between humans, tools, and environment and its impact on task performance

**Figure 2:**
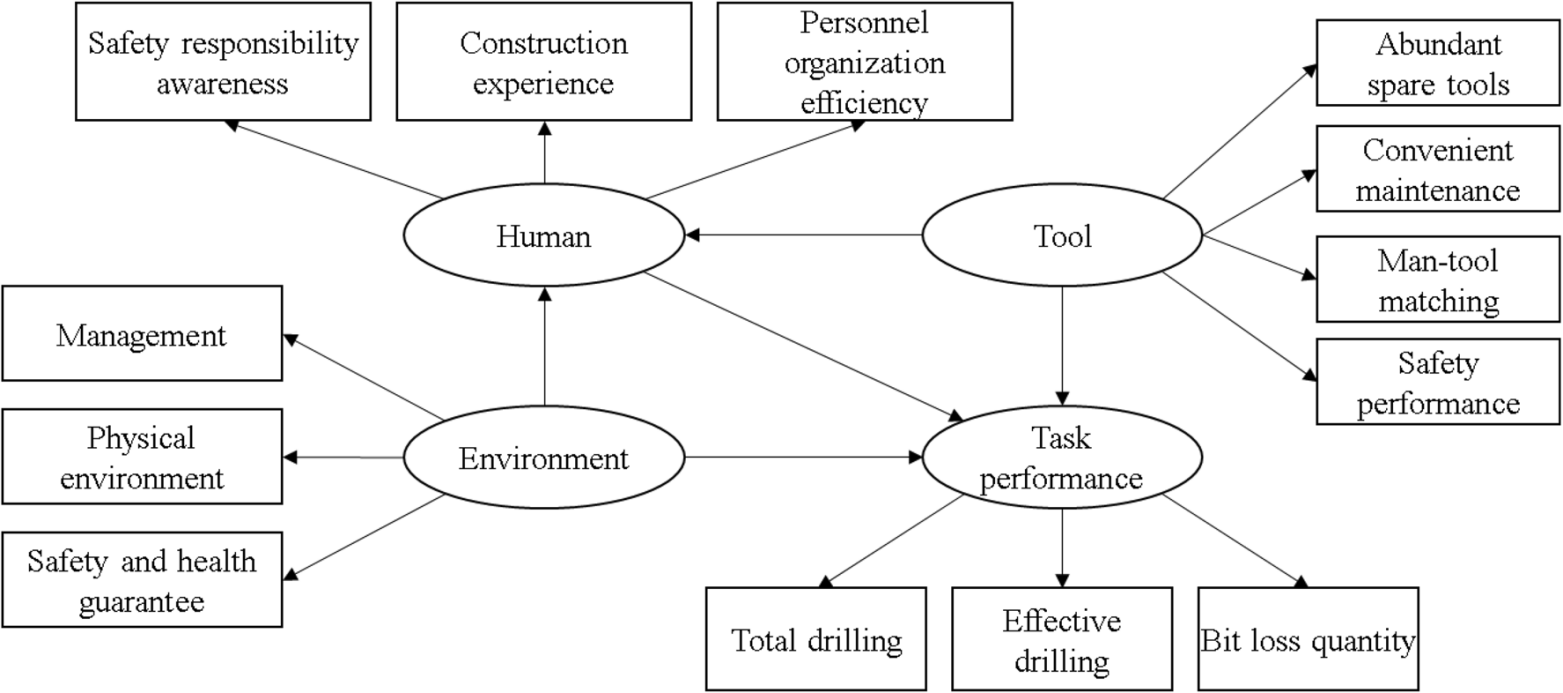
Hypothetical model diagram of relationships between influencing factors of drilling task.

Humans serve as the executors of drilling tasks, directly influencing the task performance. The quality of operational tools not only determines the drilling efficiency but also impacts the compatibility between tools and individuals, thereby affecting operational effectiveness. A favorable operating environment provides the necessary conditions for task execution and enhances the operator’s enthusiasm, whereas a poor environment can evoke negative emotions in the operator. Consequently, we developed a hypothetical relationship model (Fig. 2) that links humans, tools, the environment, and the drilling task performance. Humans, tools, and the working environment were the independent variables, the drilling performance was the dependent variable, and potential factors constituted the potential variables to explain the independent variables.

**Figure 3:**
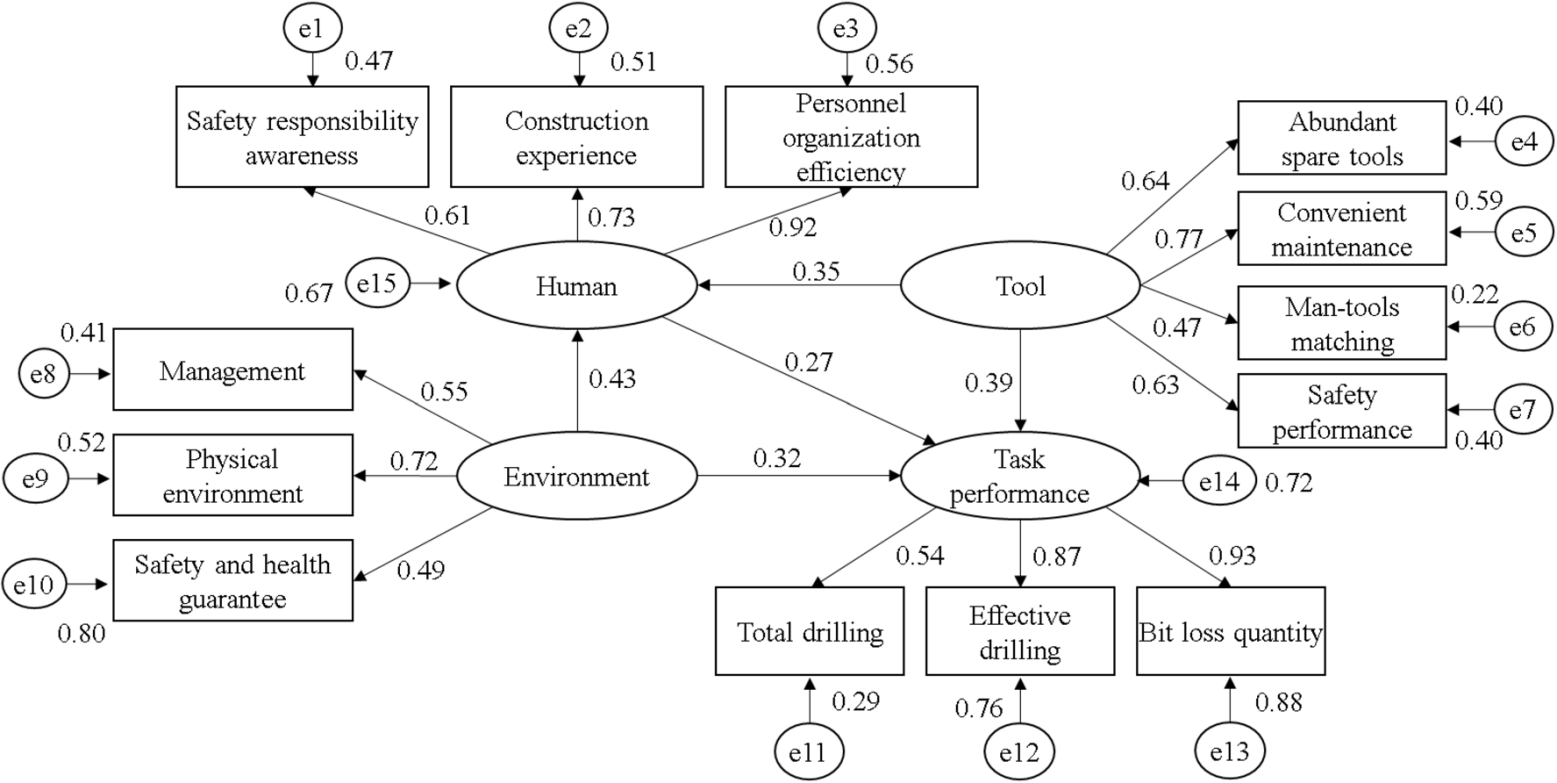
Correlation model of influencing factors of drilling task performance.

The fit indices for the survey data and hypothetical model were as follows: chi-squared = 0.428 (P ≥ 0.05), likelihood-ratio chi-squared (CMIN/DF) = 0.258, goodness-of –fit index (GFI) = 0.921, adjusted goodness-of-fit index (AGFI) = 0.936, root mean square error of approximation (RMSEA) = 0.022, non-normed fit index (NNFI) = 0.907, and comparative fit index (CFI) = 0.947. All the indices fell within an acceptable range, indicating that the hypothetical model was satisfactory. Specifically, the humans, tools, and working environment collectively accounted for 72% of the variance in the drilling task performance, while the tools and working environment could explain 67% of the variance in the human variables. The model diagram illustrating the relationship between the humans, tools, working environment, and drilling task performance is depicted in Fig. 3.

Table 5 illustrates the influence of the humans, tools, operational environment, and potential factors on the drilling task performance. The impact of potential factors on the performance was determined by multiplying the correlation coefficient between the independent variables and potential factors by the value of the total effect of the independent variables on the performance.

**Table 5:** Effect values of potential factors on drilling task performance.

Independent variable	Dependent variable	Intermediate variable	Direct effect	Indirect effect	Total effect	Potential factors	Impact of potential factors on Performance
Human	Task performance		0.27		0.27	Safety responsibility awareness	0.1647
						Construction experience	0.1971
						Personnel organization efficiency	0.2484
Tool	Task performance	Human	0.39	0.09	0.48	Availability of spare tools	0.3072
						Convenient maintenance	0.3696
						Man–tool matching	0.2256
						Safety performance	0.3024
Environment	Task performance	Human	0.32	0.12	0.43	Physical environment	0.3096
						Management system	0.2365
						Safety and health guarantee	0.2107

Based on the total effect of the independent variables on the performance, the tool variables exerted the greatest impact, followed by the operating environment variables, while human variables had the least influence on the performance. Within the tool variables, convenient maintenance, availability of spare tools, and safety performance played significant roles in the performance. For the environmental variables, the management system considerably impacted the performance. The human variables, personnel organization efficiency, and auxiliary design, as well as the environmental variables, physical environment and safety and health guarantee, had a moderate impact on the task performance. However, the safety responsibility consciousness and construction experience of operators had a relatively minor impact on the performance.

### Satisfaction evaluation of drilling task performance impact indicators

Upon comparing the disparity between the perceived performance and indicator importance, unsatisfactory indicators related to the humans, tools, and working environment were identified. To assess the significance of these differences (p < 0.05), a paired sample t-test was conducted. This test helped identify indicators whose perceived performance significantly fell below their importance. Table 6 presents the indicators where the perceived performance was notably lower than their importance.

**Table 6:** Dissatisfaction indicator and dissatisfaction degree in drilling task.

Potential factors	Indicator	Perceived performance evaluation *G*	Importance evaluation *Z*	Difference *G − Z*	P	Dissatisfaction *N*
Physical environment	Less dust	3.58	4.38	− 0.800	0.000	0.183
	Good air quality	3.38	4.28	− 0.900	0.000	0.210
	Bright light	3.50	4.58	− 1.075	0.000	0.236
	Clean construction site	3.35	4.03	− 0.675	0.000	0.169
Convenient maintenance	Sufficient vulnerable parts	3.65	4.45	− 0.800	0.000	0.180
	Easy replacement of parts	3.58	4.08	− 0.500	0.001	0.123
Safety and health guarantee	Sufficient protective materials	3.93	4.50	− 0.575	0.001	0.127
	Injury report and timely treatment	4.23	4.65	− 0.425	0.008	0.090
Personnel organization efficiency	Operation training	3.90	4.40	− 0.500	0.001	0.114
	Clear assignment of tasks	3.98	4.40	− 0.425	0.003	0.095
Availability of spare tools	Intact function and appearance	4.18	4.43	− 0.250	0.04	0.056
	Equipment types sufficient	3.90	4.38	− 0.475	0.000	0.110
Man–tool matching	Manual aided design	3.69	4.10	− 0.410	0.01	0.100

Means P < 0.05 indicates that the difference between G and Z is significant.

Among the six potential factors, 13 indicators were found to have significantly lower perceived performances than their importance, indicating dissatisfaction. The potential factors contributing to this dissatisfaction included the physical environment, convenient maintenance, safety and health guarantee, personnel organization efficiency, availability of spare tools, and man–tool matching. Higher proportions of dissatisfactory indicators within these potential factors, along with higher levels of dissatisfaction, signified a greater overall dissatisfaction. As a result, dissatisfaction levels were relatively high for the physical environment and convenient maintenance, while the dissatisfaction levels were comparatively lower for safety and health security, personnel organization efficiency, availability of spare tools, and man–tool matching.

### Priority identification of factor

**Figure 4:**
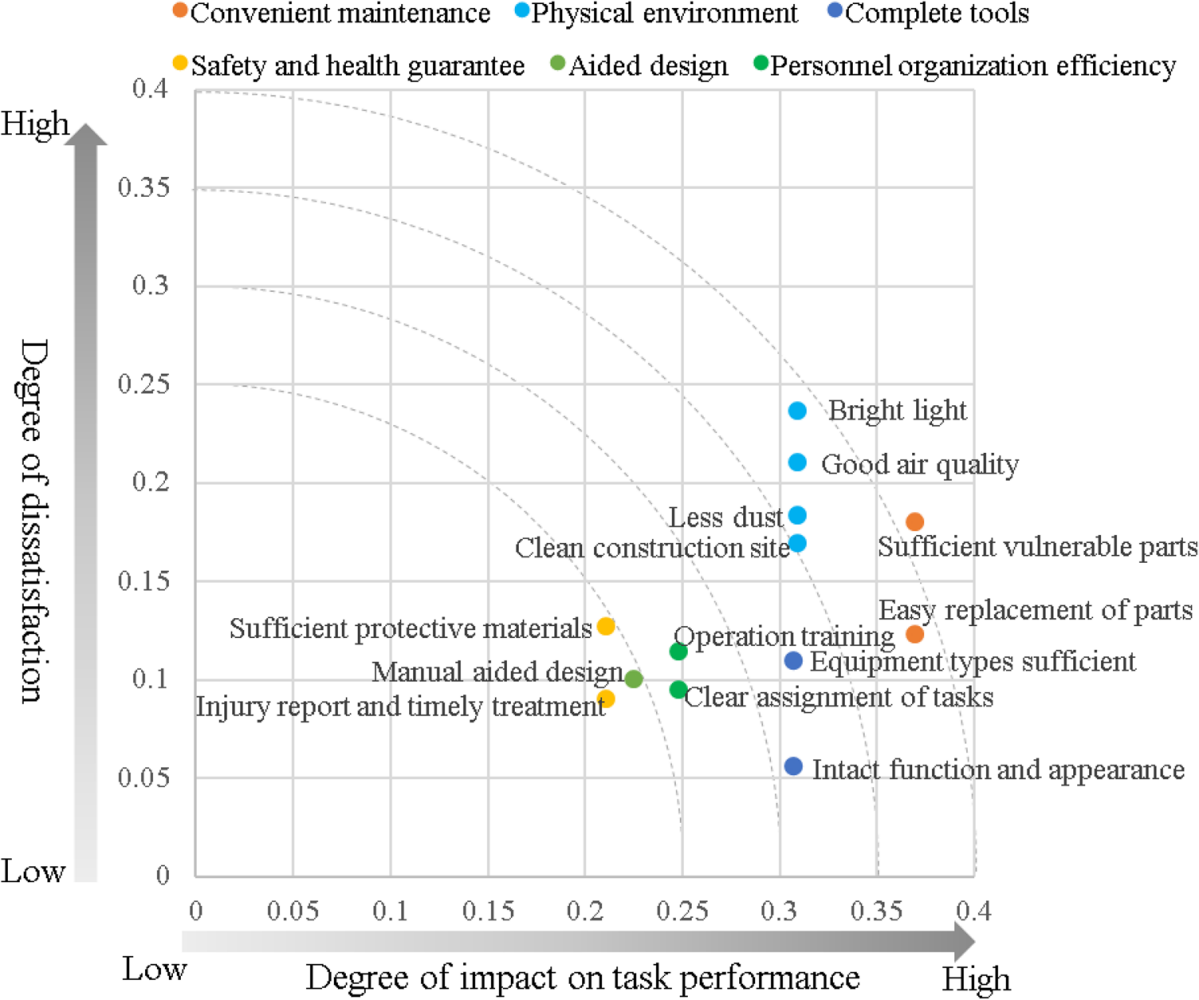
Relationship between impacts of potential factors on performance and dissatisfaction.

To optimize the drilling performance, it is crucial to consider the combined impact of potential factors on the performance and their corresponding dissatisfaction levels. This assessment helped prioritize the urgency of potential factor optimization. The primary focus should be on factors exhibiting high impact and high dissatisfaction. Figure 4 shows the sensitivity matrix. The impacts of potential factors on the performance and dissatisfaction degree were the horizontal and vertical coordinates of the matrix, respectively. A factor farther from the origin of the coordinate axis has a higher priority. Figure 4 illustrates that convenient maintenance of the tool variable had the highest importance while also having a significant degree of dissatisfaction. Therefore, improving convenient maintenance becomes pivotal in enhancing the performance of tunnel wall drilling tasks. The next priorities include enhancing the quality of the working environment and ensuring availability of spare tools, followed by improving personnel organization efficiency, refining the auxiliary design, and strengthening safety and health guarantees.

## Discussion

This study focused on investigating the key factors that affect the performance in real subway tunnel wall drilling tasks and providing essential recommendations for performance improvements. It identified indicators impacting the task performance at three levels: humans, tools, and the environment. Through factor analysis, the dimensions of these indicators were reduced, revealing potential factors influencing the performance. A structural equation model quantifies the relationships between these factors and their impact on the performance. By evaluating the satisfaction levels through a comparison of the perceived performance and importance. Key points for the performance optimization were identified based on the influence and dissatisfaction. The proposed method in this study combines the influence and dissatisfaction factors to determine the priority of optimizing the influencing factors. It offers valuable guidance on exploring the relationships between the influencing factors in complex tasks, identifying key factors, and enhancing the performance of underground space engineering construction. Additionally, this study focused on improving the task performance of subway tunnel wall drilling, which aided in improving the efficiency of subway tunnel construction and shortening the construction period.

A method is proposed to determine the priorities of the factors by combining the degree of influence and the degree of dissatisfaction. In traditional research, the importance of factors, the correlations between factors and the performance, or the degree of dissatisfaction are usually considered alone to determine the priority of the factors^42^. However, the factors with the greatest impact are not necessarily the most dissatisfactory, and the most unsatisfactory factors are not necessarily the ones with the greatest impact. For example, the results of this study showed that physical environmental factors, such as bright light and good air quality, have a great impact on the TWDTP, but they are not the most dissatisfactory factors. Convenient maintenance factors, such as sufficient vulnerable parts and easy replacement of parts, are the most dissatisfactory, but they do not have the greatest impact on the TWDTP. In this study, the sensitivity matrix was used to combine the degree of impact and the degree of dissatisfaction, and a judgment method of the factor priority was proposed. The efficacy of the sensitivity matrix was similar to that of the Kano model. The Kano model comprehensively considers two types of variables, existing-satisfaction coefficients and nonexisting-dissatisfaction coefficients, and comprehensively evaluates the priority of factors through the distance from the origin of the coordinate axes^43^.

There are also innovations in the research methods used to determine the degree of impact and the degree of dissatisfaction. When identifying the degree of impact of factors on the performance, the interrelationships between factors were considered in this study, and indirect effects were incorporated into the evaluation of the impact degree. Considering indirect effects may change the degree to which factors affect the performance and may even change the priority of factors affecting the performance^44^. For example, the inclusion of indirect influences in this study increased the difference in the degrees of impact of people, tools, and the environment on the TWDTP and made the priority of tools become more prominent. In addition, in this study, the degree of dissatisfaction was determined through differences in the perceived performance and importance^37^. In many traditional studies, the perceived performance directly represents the satisfaction of a factor. However, the results of this study show that a higher perceived performance does not necessarily represent a higher degree of satisfaction, and vice versa, which is also related to the importance of the factor. For example, the results show that, among the factors of convenient maintenance, the perceived performance of sufficient vulnerable parts was relatively higher than that of the easy replacement of parts, but the dissatisfaction level was higher, because the importance of sufficient vulnerable parts was higher, resulting in a large difference in the perceived performance and importance.

The study found that operational tools had the greatest impact on the drilling performance, followed by the environment and personnel factors. Based on the degree of dissatisfaction, the priority order of factors for improving TWDTP is convenient maintenance, the physical environment, availability of spare tools, personnel organization efficiency, man–tool matching, and safety and health guarantees.

Tool variables had the greatest impact on the TWDTP, making convenient maintenance, availability of spare tools, and man–tool matching potential targets for performance optimization. Specifically, convenient maintenance plays a crucial role in enhancing the TWDTP. These indicators reflect the high wear and failure problems of general purpose hand-held electric hammer drills in the operation of high stiffness reinforced concrete, so the ease of tool maintenance and the adequacy of spare tools become crucial. In engineering practice, the use of multi-axis robot arms with high degrees of automation has been attempted to avoid the negative impact of tools on people. However, the shock waves generated by reinforced concrete have a significant impact on the robot arm stability, resulting in damage to the robot arms. Therefore, optimizing the reliability, maintenance performance, and man–machine matching of common tools to improve their applicability in specific job tasks can help directly improve the task performance. In addition, recent studies have examined how to reduce muscle load and vibration fatigue through laboratory simulation experiments to improve work efficiency, but they did not focus on the tools, e.g. arm support exoskeletons^5,7^ and vibration protective gloves^45^. The results of this study supplement the conclusions of the simulation experiment. Specifically, while it is reasonable to consider factors that are commonly thought to be important as research topics, such as fatigue, equal attention should also be given to factors that affect task performance and contribute to worker dissatisfaction. For instance, the performance of tools becomes crucial when working with specific objects.

Physical environmental factors have a significant impact on the TWDTP, with lighting, air quality, dust, and cleanliness of the construction site being key factors for improving the TWDTP. Contrary to our concerns, the safety atmosphere in the subway tunnel wall drilling task was not shown to be important and unsatisfactory. Different from mine and tunnel excavation, the tunnel wall drilling task occurs in the later stage of the underground engineering project. Therefore, the working environment is stable and safe, and there is only a single working and operation mode. Considering the importance of designing safety climate survey items for specific industries and environments^46^, we designed safety climate survey items related to tunnel wall drilling task characteristics. Considering that the safety climate survey dimensions involved in this study were different from the general NOSACQ-50 questionnaire and the multi-dimensional items improved by Kwon et al.^46^ based on specific countries and industries, it is difficult to make a horizontal comparison with the safety climate survey results of different industries. However, it is certain that in relatively stable environments, operators using handheld tools are more concerned with the provision of favorable working conditions and the potential health and safety risks associated with their actions^26,27^. The importance of the safety climate in mining and tunnel digging industries is still important^25,28^, and according to the different stages of underground space construction tasks, it is necessary to choose different environmental factors to optimize the focus.

In tunnel drilling tasks, human factors have a relatively low impact on the task performance, although operators express dissatisfaction with personnel organization efficiency. However, in complex scenarios, human factors typically have a substantial influence on the performance^14^. Tunnel wall drilling tasks are usually carried out on a contract basis, where skilled workers work long hours to complete the task in a shorter contract time, while increasing their speed to make up for the time loss caused by factors such as equipment failures and environmental constraints. This condition leads to fatigue and health hazards, and even triggers unsafe behaviors and outcomes. Strengthening skill training and reasonable scheduling can help to improve the efficiency of personnel organization and enhance the task performance. Therefore, skill training and reasonable scheduling are necessary.

This study had several limitations. First, a survey was conducted from the perspective of workers. As a result, they may not grasp the importance of social factors, such as personnel efficiency, safety awareness, and management systems, as well as managers or scholars. This limited understanding could lead to a lower evaluation, reduced influence, dissatisfaction, or no dissatisfaction. Additionally, subway tunnel wall drilling is part of later-stage tunnel cable engineering, benefiting from favorable tunnel conditions and good safety measures. Hence, the crucial conclusion regarding optimizing operational tools may not be applicable for early-stage tunnel engineering tasks or other mining tasks with poor safety standards. Finally, the drilling task is gradually advanced with the progress of subway construction, so it is not possible to maintain a large number of drilling workers. In order to ensure the similarity of the investigation environment, the survey samples in this study were on a small scale.

To enhance the performance of tunnel wall drilling tasks, this study examined real-world drilling scenarios by a questionnaire, and identified key indicators and potential factors that affect the drilling performance. By investigating the influence and dissatisfaction of these factors on the task performance, it was determined that the operation tool had the greatest impact on the drilling performance, followed by environmental quality, while human factors had the least influence. Furthermore, among the 13 indicators related to these factors, convenient maintenance of tools significantly affected the TWDTP and yielded high dissatisfaction, making it a crucial area for improvement. In addition, improving the quality of the physical environment is important. Additionally, ensuring that there is availability of spare tools, enhancing personnel organization efficiency, optimizing auxiliary design, and strengthening the safety and health measures are vital. This study established a priority order for optimizing influencing factors to enhance the tunnel wall drilling task performance. It also provided recommendations for improving the human–machine environmental system in tunnel drilling tasks, thereby benefiting manual operations during later stages of tunnel construction.

## Data Availability

The datasets generated and analyzed during the current study are available from the corresponding author upon reasonable request.
